# Early Blood–Brain Barrier Impairment as a Pathological Hallmark in a Novel Model of Closed-Head Concussive Brain Injury (CBI) in Mice

**DOI:** 10.3390/ijms25094837

**Published:** 2024-04-29

**Authors:** Stefan J. Blaschke, Nora Rautenberg, Heike Endepols, Aileen Jendro, Jens Konrad, Susan Vlachakis, Dirk Wiedermann, Michael Schroeter, Bernd Hoffmann, Rudolf Merkel, Niklas Marklund, Gereon R. Fink, Maria A. Rueger

**Affiliations:** 1 Department of Neurology, Faculty of Medicine and University Hospital, University of Cologne, 50923 Cologne, Germany; nora.rautenberg@uk-koeln.de (N.R.); ajendro@smail.uni-koeln.de (A.J.); michael.schroeter@uk-koeln.de (M.S.); gereon.fink@uk-koeln.de (G.R.F.); maria.rueger@uk-koeln.de (M.A.R.); 2 Cognitive Neuroscience Section, Institute of Neuroscience and Medicine (INM-3), Research Centre Juelich, 52428 Juelich, Germany; 3 Institute of Radiochemistry and Experimental Molecular Imaging, Faculty of Medicine and University Hospital Cologne, University of Cologne, 50937 Cologne, Germany; hendepo1@uni-koeln.de; 4 Department of Nuclear Medicine, Faculty of Medicine and University Hospital Cologne, University of Cologne, 50931 Cologne, Germany; 5 Nuclear Chemistry, Institute of Neuroscience and Medicine (INM-5), Research Centre Juelich, 52428 Juelich, Germany; 6 Mechanobiology, Institute of Biological Information Processing (IBI-2), Research Centre Juelich, 52425 Juelich, Germany; j.konrad@fz-juelich.de (J.K.); b.hoffmann@fz-juelich.de (B.H.); r.merkel@fz-juelich.de (R.M.); 7 Multimodal Imaging Group, Max Planck Institute for Metabolism Research, 50931 Cologne, Germany; dirk.wiedermann@sf.mpg.de; 8 Department of Clinical Sciences Lund, Neurosurgery, Lund University, 221 85 Lund, Sweden; niklas.marklund@med.lu.se

**Keywords:** mild traumatic brain injury, mice, repetitive concussion, blood–brain barrier, neuroinflammation, PET

## Abstract

Concussion, caused by a rotational acceleration/deceleration injury mild enough to avoid structural brain damage, is insufficiently captured in recent preclinical models, hampering the relation of pathophysiological findings on the cellular level to functional and behavioral deficits. We here describe a novel model of unrestrained, single vs. repetitive concussive brain injury (CBI) in male C56Bl/6j mice. Longitudinal behavioral assessments were conducted for up to seven days afterward, alongside the evaluation of structural cerebral integrity by in vivo magnetic resonance imaging (MRI, 9.4 T), and validated ex vivo by histology. Blood–brain barrier (BBB) integrity was analyzed by means of fluorescent dextran- as well as immunoglobulin G (IgG) extravasation, and neuroinflammatory processes were characterized both in vivo by positron emission tomography (PET) using [^18^F]DPA-714 and ex vivo using immunohistochemistry. While a single CBI resulted in a defined, subacute neuropsychiatric phenotype, longitudinal cognitive testing revealed a marked decrease in spatial cognition, most pronounced in mice subjected to CBI at high frequency (every 48 h). Functional deficits were correlated to a parallel disruption of the BBB, (R^2^ = 0.29, *p* < 0.01), even detectable by a significant increase in hippocampal uptake of [^18^F]DPA-714, which was not due to activation of microglia, as confirmed immunohistochemically. Featuring a mild but widespread disruption of the BBB without evidence of macroscopic damage, this model induces a characteristic neuro-psychiatric phenotype that correlates to the degree of BBB disruption. Based on these findings, the BBB may function as both a biomarker of CBI severity and as a potential treatment target to improve recovery from concussion.

## 1. Introduction

Traumatic brain injury (TBI) remains a common and potentially devastating disease, especially impacting younger populations, with a high degree of mortality and morbidity [1]. The past decades have highlighted the multifaceted pathophysiological spectrum of TBI [2]. The mildest form of TBI—the mere concussion—caused by a rotational acceleration/deceleration injury, occurs most frequently, and by definition, it does not cause any macroscopic structural brain damage [3]. However, in recent years, it has become increasingly obvious that concussions—especially when they occur repeatedly as commonly observed in contact sports—may induce concussive brain injury (CBI) with a substantial impact on life and well-being, particularly in the juvenile and young adult group [4]. Accumulating evidence suggests that post-concussive symptoms (i.e., chronic headaches, dizziness, or impairment of memory and concentration) not only persist in a subgroup of patients over weeks to months (i.e., the “miserable minority” [5,6]) but may also lead to secondary deterioration due to accelerated neurodegeneration, especially after repetitive trauma [7,8]. Since conventional neuroimaging does not detect structural abnormalities after a concussion, CBI and its related persistent post-concussion syndrome are not easily diagnosed, and most healthcare providers are unsure about its prognosis, leading to uncertainty in both patients and healthcare providers [9]. Consequently, persistent symptoms after CBI might be trivialized or suspected as malingering [10], leading to inadequate medical care and chronification. Thus, while a diagnostic arsenal is urgently needed to diagnose CBI and estimate an individual prognosis for CBI patients, the underlying pathophysiological hallmarks and the (variable) temporal course of CBI remain poorly understood.

To uncover underlying disease mechanisms, preclinical models of various degrees of TBI have been assessed. Nonetheless, there is an astonishing lack of animal models for the characteristic CBI injury mechanism. Most often, a mild form of TBI is induced by a focal blow to the fixated head of the experimental animal, with scaled mechanical intensity [11]. However, this does not exactly reflect the situation of, e.g., the impact on the head when boxing or playing soccer (header), since a blow to the unfixated head causes its rotation [12]—therefore inducing a more diffuse and multifocal brain trauma while avoiding skull fractures [11].

Among the few preclinical models of rotational closed-head injury described to date, a heterogeneous pathophysiology is highlighted, mainly featuring neuroinflammation [13,14], blood–brain barrier (BBB) disruption, and microvascular injury [15,16], as well as electrophysiological alterations [16,17]. To date, pathophysiological findings on the focal/cellular level have not been comprehensively correlated to functional/behavioral deficits in the respective animal models.

We here aimed to characterize a novel model of CBI featuring a mild rotational brain injury in the mouse. To alleviate the need for specific biomarkers of disease severity and biological recovery, we specifically assessed the effects of CBI on BBB disruption and neuroinflammation. In this way, we specifically evaluated a second-generation translocator protein (TSPO) tracer, [^18^F]DPA-714, as a surrogate marker of secondary brain injury, corroborating in vivo data by ex vivo histology and correlating those parameters to the disease severity of the individual behavioral phenotype.

## 2. Results

### 2.1. Novel Mouse Model of Rotational Closed-Head Concussive Brain Injury (CBI)

We aimed to establish a highly reproducible mouse model of CBI that resembles key hallmarks of concussion observed in humans. Thus, we applied (i) an external force on the (ii) freely moving (iii) closed skull in (iv) a controlled fashion that (v) induced a rotational acceleration–deceleration force on the brain, (vi) without causing structural lesions such as brain contusion or bone fractures.

Additionally, to prevent the presumably (neuro-)protective effect of anesthesia [18] and preserve neuronal activity at the timepoint of injury, we induced CBI in conscious sedation with medetomidine, a drug widely used to preserve functional network activity in neuroimaging [19].

**Figure 1:**
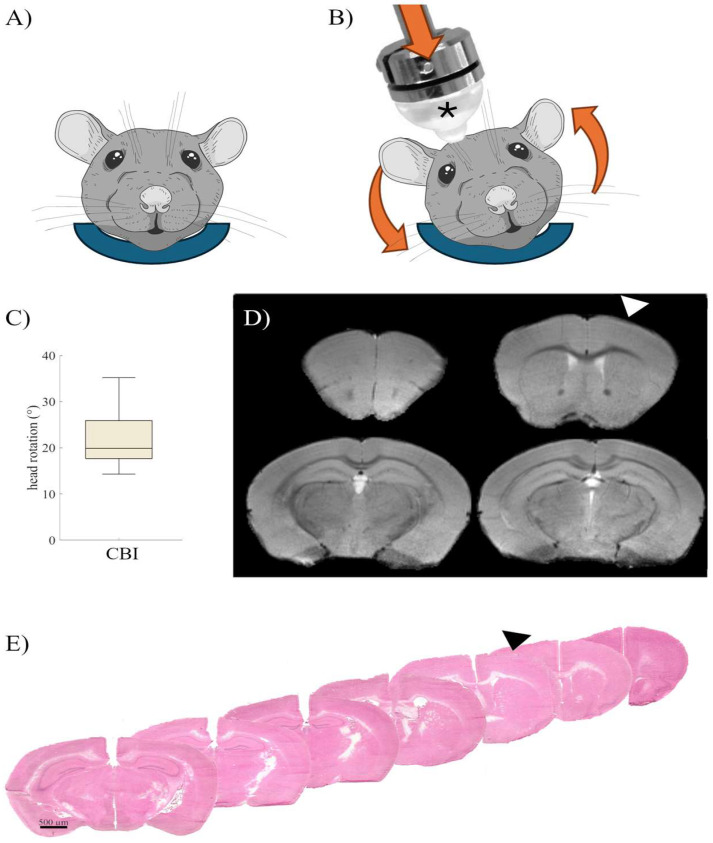
To induce the closed-head acceleration–deceleration concussive brain injury (CBI), mice were placed in a foam bed ((**A**) blue). The head was struck by an electromagnetically driven impactor, softened with a custom-made 5 mm silicon tip ((**B**), *). Thus, the head, limiting linear translation by the foam bed, was rotated counterclockwise in the coronal plane by about 20° (**C**). As assessed by early structural MRI ((**D**) T2 weighted TurboRARE), even repetitive concussions at high frequency (repCBI-hf) did not cause cerebral contusions or microbleeds, as additionally verified by hematoxylin and eosin staining ((**E**) impact location indicated by the arrowhead).

Inducing the concussion, we observed a reproducible head rotation (Figure 1C), with only minor variations depending on the exact position of the mouse head (mean rotation 22°, SD = 6.7°). The impact parameters were chosen based on previous finite element analyses in mice [20] to reproduce similar biomechanical forces as in human CBI. To mitigate the focal strain on the skull and the brain, we used a rounded impactor tip that was additionally softened by a custom-made silicon cap. Hitting the skull with this tip at 5 mm/s did not cause a skull fracture in any of the mice, while a higher impactor speed of 6 mm/s led to occasional skull fractures. Most noteworthy, the assessment of the structural integrity of the brain one day after the last concussion, using in vivo T2-weighted MRI (Figure 1D) as well as ex vivo HE-staining (Figure 1E), indicated no signs of macroscopic tissue disruption, contusions, or microbleeds in any of the mice.

### 2.2. CBI Induces a Neuro-Psychiatric Phenotype

**Figure 2:**
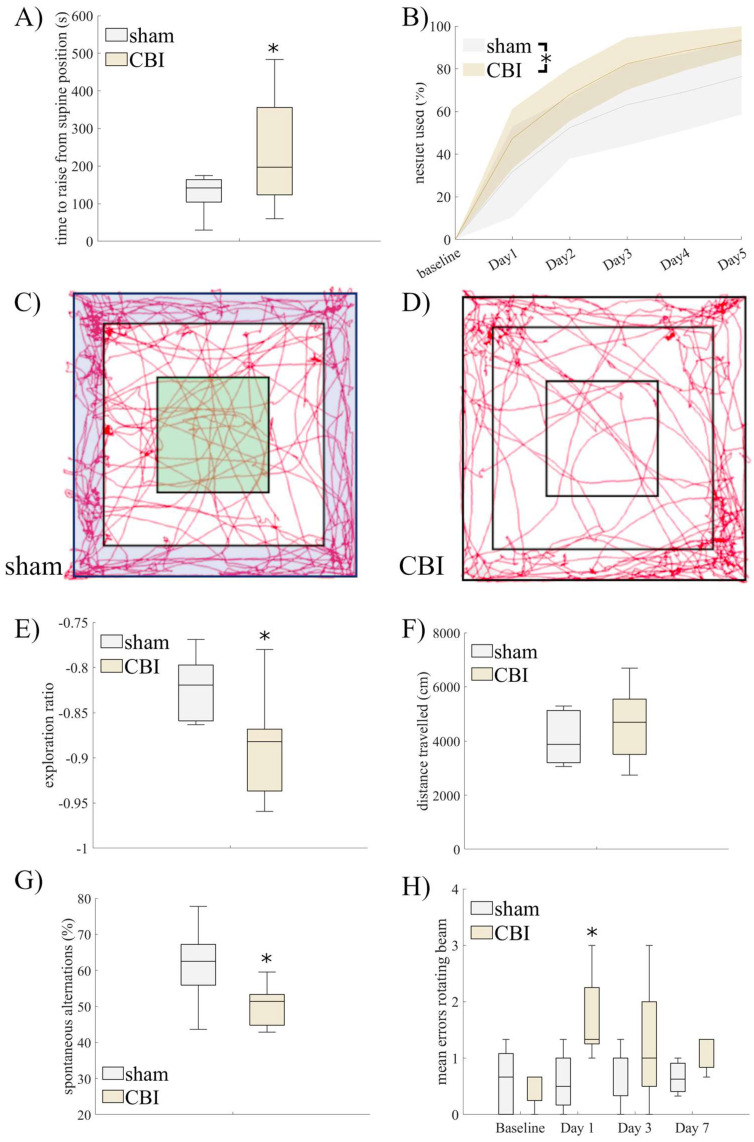
A single CBI induced a significantly longer latency to rise from a supine position after the trauma (**A**). Over the first days after CBI, animals exhibited significantly higher overall activity levels (**B**), as measured by the amount of nesting materials used over time. In the Open Field, relative exploration of the center zone ((**C**), green) was compared to exploration of the outer border zone (blue). While healthy (sham) animals (**C**) explored all regions evenly, CBI mice (**D**) showed a significant decrease in exploration of the center zone and instead kept to the walls of the arena, suggesting anxiety (**E**), while their overall locomotor activity was not reduced (**F**). Mice suffering from CBI displayed a significant decrease in spatial cognition in the spontaneous alternation test (Y-maze, (**G**)) and a transient lack of motor control (rotating beam, (**H**)). (* *p* < 0.05 compared with sham, n (sham) = 6, n (CBI) = 9).

We next characterized the clinical phenotype of our novel CBI model. Using a surrogate parameter for CBI-induced loss of consciousness (LoC), animals receiving a concussion required a significantly longer time to regain full alertness after this procedure and rise from a supine position (Figure 2A, 234 s, [171, 297]) compared with the sham group (127 s, [89, 165]; U = 4.6, *p* = 0.03). Over the next five days, mice suffering from CBI displayed a higher overall activity level, as indicated by their extensive use of nestlet material (Figure 2B, CBI: mean AUC = 63.2, [56.9, 69.5], sham: mean AUC = 48.8, [36.2, 61.4]; U = 6.6, *p* = 0.01). In the Open Field, the control (sham) group of mice evenly explored the entire arena (Figure 2C; exploration ratio = −0.82, [−0.87, −0.78]), while mice suffering from CBI kept moving along its walls and avoiding the center zone, suggesting increased anxiety (Figure 2D,E; exploration ratio = −0.89, [−0.93, −0.85], U = 5.4, *p* = 0.02). Consistent with the result on overall motor activity, the overall distance traveled by CBI mice was not significantly decreased (CBI: mean distance = 4.6 m, [3.6, 5.6]; sham: 4.1, [2.8, 5.4]), despite their avoidance of the center zone (Figure 2F).

On the level of spatial cognition, mice underperformed in the Y-maze test after CBI (48.7, [41.7, 55.7]) compared with healthy (sham) animals (Figure 2G; 61.5, [46.4, 76.6]; U = 4.7, *p* = 0.04). Cognitive impairment assessed as spontaneous alternations in the Y-maze was corroborated by similar results in the Novel Object Recognition (NOR) Test, albeit with a non-significant decrease in the recognition rate after CBI (0.26, [0.12, 0.39]; U = 2.9, *p* = 0.08; Figure S1B) compared with the sham group (0.51, [0.06, 0.97]). However, there was a significant correlation between individual scores in the Y-maze and NOR Test (R^2^ = 0.65, *p* < 0.01, Figure S1C). Motor control as assessed by the rotating beam test revealed a mild motor impairment one day after CBI (Figure 2H). That impairment spontaneously ameliorated over the first week after a concussion, indicated by a significant main effect of the trauma group assignment (F(1,40) = 6.0, *p* = 0.02) and a significant difference between groups one day after injury in the simple effects analysis.

### 2.3. CBI Leads to Sustained Impairment in Spatial Cognition

**Figure 3:**
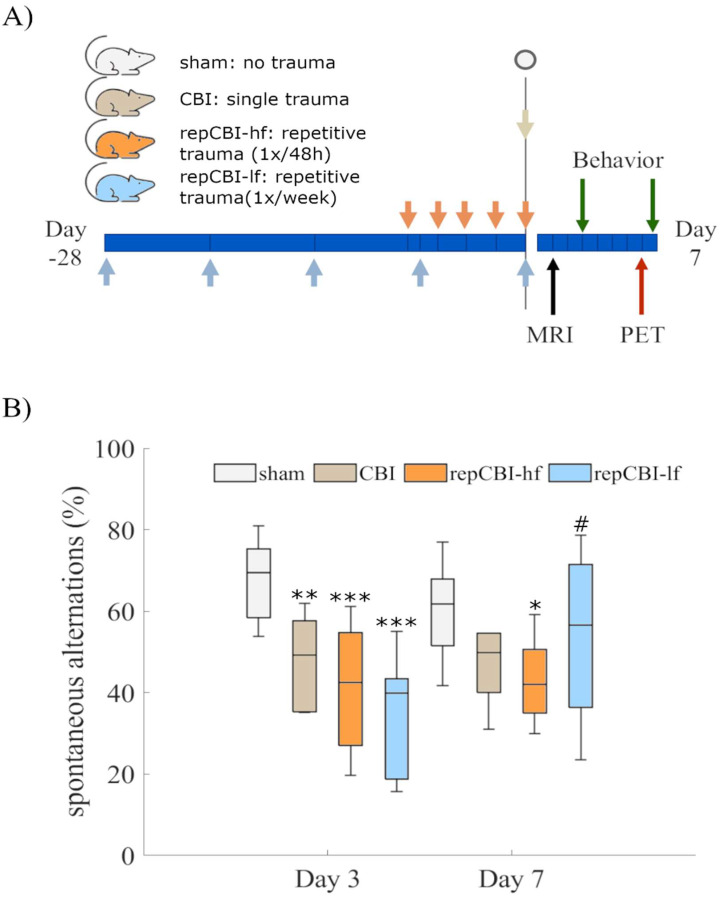
(**A**) To characterize the effects of repetitive CBI, mice received five consecutive concussions either every 48 h (repCBI-hf) or once a week (repCBI-lf) and were compared to a group of mice receiving a single CBI or sham surgery only. (**B**) CBI induced a significant decrease in spatial cognition in all groups compared with sham three days after the last trauma. However, seven days after (the last) CBI, cognitive impairment was still visible only after a single CBI (light grey), as well as after concussions delivered at a high frequency (orange), while the low-frequency CBI group (blue) improved most rapidly. (* *p* < 0.05; ** *p* < 0.01, ***p < 0.001 compared with sham, # *p* < 0.05 compared to day 3, n = 10).

In order to characterize the effects of repetitive CBI, groups of mice were subjected to several concussions in different intervals, leading to different periods of recovery between impacts (Figure 3A). Five consecutive concussions, either (i) every 48 h (repCBI-hf, orange) or (ii) every week (repCBI-lf, blue), were compared to (iii) a one-time CBI (CBI, light grey), and to (iv) sham-injured healthy mice (sham, white; Figure 3A).

Based on spontaneous alternations in the Y-maze test, a mixed ANOVA with repeated measures over time and trauma as a between-group factor, revealed a significant effect of trauma on spatial cognition (F(3,24) = 7.9, *p* < 0.001), with a significant interaction effect of time and trauma (F(3,24) = 5.8, *p* = 0.004). Mice receiving a single CBI exhibited a subacute impairment in spatial cognition after three days (CBI: 48.1, [36.2, 59.9], sham: 67.7, [59.3, 76.0]), which slightly improved after seven days (Figure 3B, CBI: 46.6, [32.0, 75.7], sham: 60.2, [50.5, 69.8]). A particularly long-lasting decrease in spatial cognition was observed in the group of mice suffering from repetitive CBI at short (48 h) intervals (repCBI-hf, Figure 3B; Day 3: 41.2, [28.2, 54.1], Day 7: 43.1, [34.5, 51.6]). Interestingly, mice subjected to repetitive CBI at weekly intervals—allowing longer recovery phases between concussions—recovered most rapidly from their last concussion, reaching mostly their baseline cognitive level 7 days after their last concussion (repCBI-lf, Figure 3B, Day 3: 35.4, [19.3, 51.5], Day 7: 53.9, [32.0, 75.7]).

### 2.4. Repetitive CBI Causes Lasting Disruption of the Blood–Brain Barrier (BBB)

**Figure 4:**
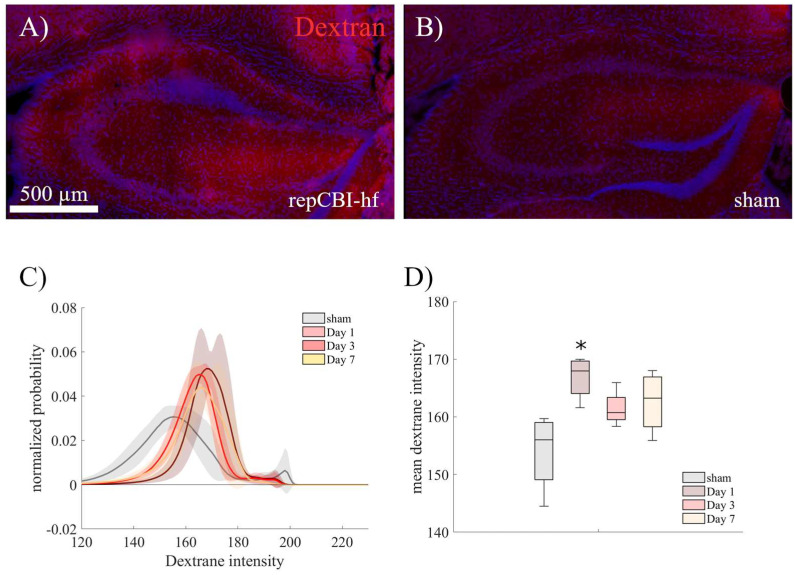
TxRed-labeled dextran (40 kDa) was infused one, three, or seven days after repetitive CBI at high frequency to evaluate the effects of CBI on the temporal dynamics of blood–brain barrier impairment. CBI animals showed a significant dye leakage one day (**A**) after the last concussion compared with sham animals (**B**). This effect was detectable over the first week after CBI (**C**,**D**). (* *p* < 0.05 compared with sham, n = 4).

The dynamic effect of repetitive CBI on the integrity of the BBB over time was evaluated by infusion of TxRed-labeled dextran during the terminal perfusion of mice. One day after a set of five concussions every 48 h, dye leakage—causing a blurry fluorescent signal—appeared most prominent in the hippocampus (Figure 4A) compared with sham mice, where the dextran signal was located intravascularly for the most part (Figure 4B). Quantification of this observation based on the fluorescence intensity of labeled dextran (Figure 4C) demonstrated a significant effect of trauma (H(3,15) = 9.3, *p* = 0.026) with the largest effect of CBI on BBB integrity one day after the (last) trauma (mean intensity Day 1: 38.1, [32.0, 44.2], sham: 51.0, [40.0, 61.9]; *p* = 0.012), with incomplete recovery over the course of seven days (Figure 4D; mean intensity Day 7: 42.4, [33.7, 51.1]).

### 2.5. CBI-Induced Impairment of the BBB Is Linked to the Behavioral Phenotype

We next characterized the region-specific extent of BBB disruption to evaluate an interrelationship with brain function. Therefore, we used the extravasation of (endogenous) immunoglobulins (IgG) as a surrogate parameter for BBB dysfunction one week after the (last) concussion.

**Figure 5:**
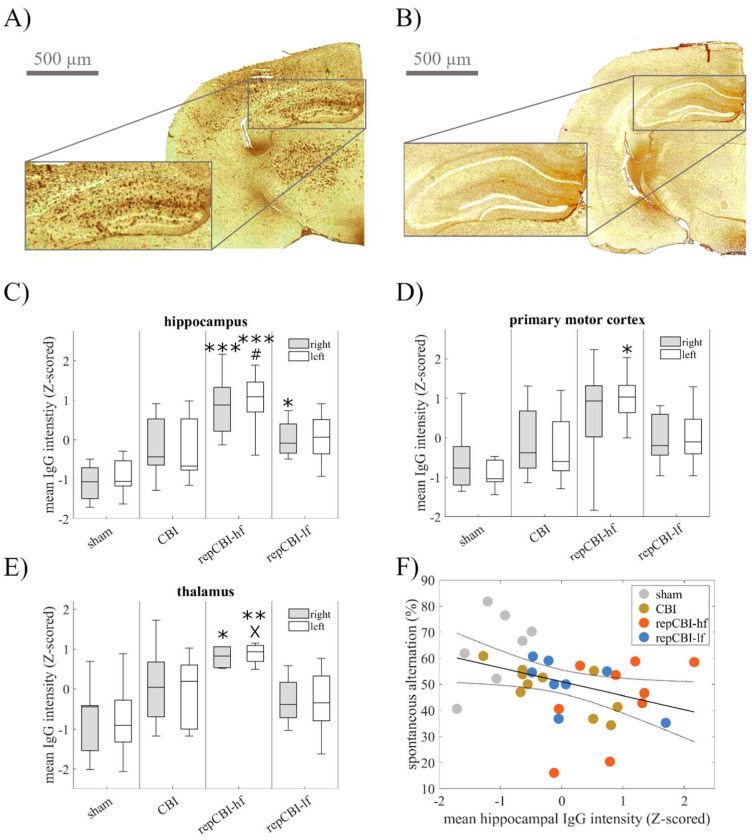
Disruption of the BBB one week after the last concussion was quantified via extravasation of (endogenous) immunoglobulins (IgG) as a surrogate parameter. Compared with sham animals (**A**), mice subjected to repetitive CBI showed a profound and multilocular impairment in BBB function (**B**), especially within the lateral cortex, hippocampus, and thalamus of both hemispheres. This effect was most pronounced after repetitive CBI in high frequency, prominently affecting the brain regions responsible for the behavioral impairment detailed above: hippocampus (**C**), thalamus (**D**), and primary motor cortex (**E**). Consequently, levels of IgG extravasation correlated with impairment in spatial cognition (**F**). (* *p* < 0.05; ** *p* < 0.01; *** *p* < 0.001 compared with sham, # *p* < 0.05 compared with mTBI, X *p* < 0.05 compared with rmTBI-low; n [sham, CBI, rmCBI-hf, rmCBI-lf] = 8, 10, 9, 8).

Corroborating the results obtained with dextran (cmp. Figure 4), mice subjected to repetitive CBI (Figure 5A) showed a profound and multilocular impairment of the BBB compared with sham animals (Figure 5B). This effect was most pronounced after repetitive CBI in high frequency, i.e., five consecutive concussions every 48 h (Figure 5C–E). Most interestingly, brain regions implied in the behavioral impairments detailed above (cmp. Figure 2 and Figure 3) were most prominently affected. In particular, we saw a significant BBB disruption in the hippocampus (Figure 5C), in the primary motor cortex (Figure 5D), and in the thalamus (Figure 5E). In particular, levels of IgG extravasation in the hippocampus correlated well with the impairment in spatial cognition as assessed by the Y-maze test (R^2^ = 0.18, *p* = 0.01, Spearman correlation, Figure 5F).

### 2.6. Detection of Neuroinflammation by Positron Emission Tomography (PET) Is Distorted by BBB Disruption

**Figure 6:**
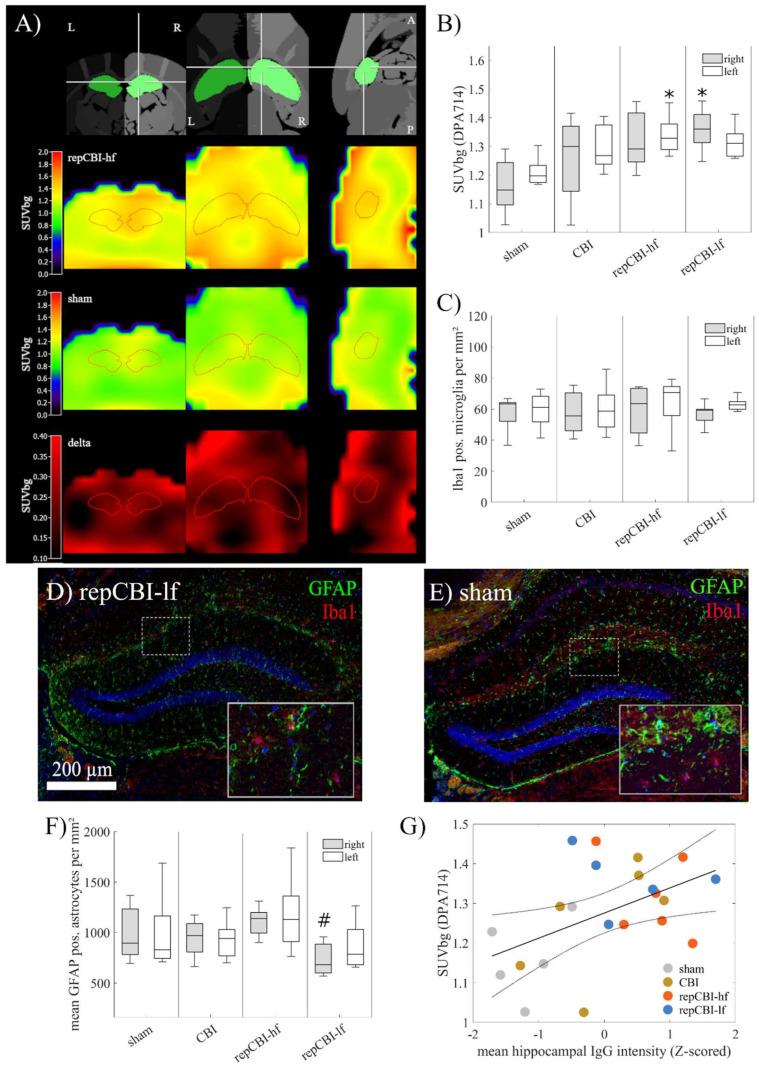
(**A**) Second-generation TSPO tracer [^18^F]DPA714 showed an increased uptake, especially after repetitive CBI (intensity normalization to background signal, R = right, L = left, A = anterior, P = posterior), with a significant bilateral increase in hippocampal uptake (**B**) one week after (the last) concussion (n = 6). However, at this time, radiotracer accumulation was not explained by elevated signs of activated Iba-1+ microglia (**C**), evaluated after single or repetitive CBI (**D**) compared to the sham group (**E**). Likewise, the amount of GFAP+ reactive astrocytes (**F**) in the hippocampus was not significantly altered compared to the sham group, with a significant difference between the groups of repetitive trauma (n [sham, CBI, rmCBI-hf, rmCBI-lf] = 8, 10, 9, 8). Thus, [^18^F]DPA714 accumulation was potentially explained by the disturbance of the BBB one week after concussion, with a good correlation between tracer uptake and IgG extravasation in the hippocampus (**G**). (* *p* < 0.05 compared with sham, # *p* < 0.05 compared with repCBI-hf).

In order to evaluate neuroinflammatory processes following CBI, we used positron emission tomography (PET) and the second-generation TSPO tracer [^18^F]DPA714 to detect and quantify immune cell activation. We indeed observed an increased uptake of [^18^F]DPA714 (Figure 6A), with significantly elevated standard uptake values (SUVs), especially in the hippocampus after repetitive CBI, one week after the last concussion (Figure 6B).

However, immunohistological analyses revealed that tracer uptake was not due to an increase in activated Iba-1+ microglia (Figure 6C), as quantified in the hippocampus in either group of mice (Figure 6D,E). While we found a significant group effect of trauma on astrocyte density (H(3,33) = 9.5, *p* = 0.02, Figure 6F), the effect was not due to a difference compared to sham injured animals (99.2 cells/mm^2^, [75.2, 123.3]) but a significant decrease in astrocyte density after low-frequency repetitive CBI (repCBI-lf: 73.3 cells/mm^2^, [60.2, 86.4]) compared with the high-frequency group (repCBI-hf: 104.5 cells/mm^2^, [81.6, 127.5]; *p* = 0.01). On the other hand, [^18^F]DPA714 radiotracer accumulation correlated well with the extent of BBB disruption, as assessed by IgG extravasation (R^2^ = 0.21, *p* = 0.03, Spearman correlation, Figure 6G), suggesting unspecific tracer accumulation as a result of increased BBB permeability. However, further analyses of the time–activity curves did not show a difference in the temporal tracer dynamics (Figure S1I), warranting further investigation.

## 3. Discussion

We here characterize a novel mouse model of CBI, featuring a rotational brain injury without macroscopic structural damage. Our model reveals a characteristic neuro-psychiatric phenotype resembling CBI in patients or athletes that correlates to the amount of BBB disruption, thus suggesting a biomarker for disease severity and, potentially, for disease prognosis in the future.

Mice subjected to our novel CBI model display a hypervigilant phenotype, with increased anxiety, a sustained impairment in spatial cognition, and a mild and short-lived disturbance of motor control. This phenotype resembles part of the clinical spectrum in patients with CBI [5,6]. Interestingly, we saw a faster recovery of spatial cognition in animals subjected to weekly concussions, both in comparison with mice suffering from concussions at a higher frequency (every 48 h), even compared to mice subjected to only a single (one-time) concussion. This suggests a pre-conditioning effect of preceding traumata [21], but only when sufficient time for recovery was granted between concussions. This finding has high relevance for sports-associated concussions, as it may impact current return-to-play guidelines. Similar to our findings, in an alternative mouse model of non-rotational TBI induced by a weight drop, faster recovery of cognitive function occurred as assessed by Morris Water Maze, when mice were exposed to TBI at monthly intervals as compared with higher TBI frequencies [22].

As a key finding on the structural level, we here report a robust and multifocal BBB breakdown in the subacute phase after CBI, which was most pronounced after repetitive concussions. BBB integrity plays an important role in maintaining a healthy neural state, while disruption of the BBB constitutes a known driver of disease progression, e.g., in ischemic stroke, multiple sclerosis, or neurodegenerative disease [23]. While widely observed after all forms of TBI [24], the role of BBB impairment in the development of functional/behavioral deficits as well as in the recovery of function remains to be established. Various preclinical models of mild TBI have reported disturbance of the BBB, e.g., in swine [25], rats [26], and mice [27]. Moreover, indirect evidence by means of gadolinium contrast extravasation on MR-imaging was observed in repetitive sports-associated concussions in humans [28].

By means of both the infusion of labeled dextran as well as staining for IgG extravasation, we here describe an ongoing disruption of the BBB even seven days after CBI. On the same note, ongoing impairment of the BBB, detectable hours after trauma [29], was described for at least days [30,31,32]—in one study, for up to two weeks [33]—after repetitive trauma in various preclinical models. Using laser speckle imaging, Lynch et al. demonstrated impairment in cerebrovascular reactivity several months after repetitive mild TBI [34]. Corresponding to our result on the effect of CBI frequency on the extent of BBB damage (cmp. Figure 5), intraindividual longitudinal non-invasive monitoring of BBB function by contrast-enhanced MRI over the course of three mild head impacts in rats revealed an increasing degree of BBB permeability [35]. While most murine closed-head injury models highlight some kind of TBI-induced BBB alterations [36], Wu et al. described a model of rotational acceleration–deceleration in the sagittal plane, explicitly lacking Evans blue extravasation or IgG leakage from vessels [37]. Although considerable differences in the mechanical parameters used for trauma induction render a comparative interpretation difficult, these divergences suggest a relevance of the direction of head rotation for the type of brain injury induced.

In contrast to the robust BBB breakdown, we did not observe a significant alteration in astrocyte density within the hippocampus compared to sham-injured animals. Of note, we noted a slight difference in astrocyte density across CBI groups. While astrocyte density was reduced in the group of animals receiving CBI at low (weekly) frequency, it was considerably higher in mice subjected to CBI every 48 h. We argue that on top of a potential mechanical disruption of the neurovascular interface, CBI might induce relevant reactive astrocytosis only at high but not at low frequency. A recent study reported an atypical astrocytic response in the weight-drop model of mild TBI in areas of sustained BBB impairment that was detectable for months after the trauma [27]. Otherwise, glial dysfunction with reduced hippocampal GFAP expression levels was previously reported in preclinical depression models of chronic psychosocial stress [38]. Overall, further analyses of the temporal and molecular underpinnings of the effects of CBI on astrocyte function are certainly warranted.

The tightly regulated dynamic BBB serves as a core component of the neurovascular unit that is intimately involved in neuroinflammatory processes, by leaking neurotoxic products as well as hematogeneous pro-inflammatory immune cells into the brain, while, at the same time, being disrupted by neuroinflammatory mediators in a reciprocal fashion [39]. Thus, after TBI, BBB impairment may originate (primarily) from mechanical disruption of capillaries, and then be maintained (secondarily) by persistent neuroinflammation. However, recent evidence from preclinical models of mild TBI, e.g., in rats [26] or swine [25], highlights BBB impairment as a key pathological hallmark even in the absence of neuroinflammation, indicating the existence of complementary mechanisms. Astrocytic activation may be induced by the trauma itself as well as a secondary phenomenon to protein extravasation, change in neurological activity [40], rise of extracellular potassium, and spreading depression [41,42].

Chronic and aberrant neuroinflammation is a hallmark of many neurological disorders without a primary immune-mediated etiology, such as stroke, Parkinson’s disease, Alzheimer’s disease, and epilepsy, rendering it a promising future therapeutic target [43,44]. In moderate to severe TBI leading to structural brain damage, neuroinflammatory processes have been well characterized, even in the (sterile) setting without injury to the dura mater [45]. However, the presence and time course of neuroinflammation after pure concussion—without macroscopic structural lesions to the brain—remains a matter of constant debate [46]. We and others have previously shown that brain-endogenous immune cells, i.e., microglia, respond to even subtle mechanical cues, e.g., stretch [47] or mere tissue elasticity [48], and thus hypothesized that microglia activation would be seen in CBI. A few studies observing microglia activation after mild TBI/CBI described simultaneous neuronal injury/axonal degeneration, even if detectable only ex vivo [15,49], or did not perform histological evaluation of structural damage [50]. Our novel CBI model did not cause any structural lesion detectable in vivo (MRI) or ex vivo (histologically) and also found no evidence of activated microglia, albeit observing the accumulation of the second-generation TSPO radiotracer [^18^F]DPA-714 one week after CBI. Ligands to the 18 kDa translocator protein TSPO, an outer membrane molecule highly upregulated in microglia, have been widely used to monitor neuroinflammation in, e.g., stroke [51] or neurodegeneration [52], but other sources of TSPO accumulation, e.g., increased neuronal activity [53], reactive astrocytes [54], or endothelial cells [55], have been debated to contribute to the PET signal. We here observed radiotracer accumulation to coincide with BBB disruption, but a longitudinal study with different time points of PET-imaging (including kinetic modeling) would be required to verify a causal connection. TSPO accumulation due to BBB disruption may overcast more subtle and putatively transient microglia activation. Recently, TSPO has been linked to microglia proliferation and increased density in CNS pathology but not an activated state of microglia state per se [56,57]. Histological analyses of higher temporal granularity will be needed to deeper elucidate the astro- and microglial response to our novel model of CBI.

Altogether, the present findings highlight the potential of blood–brain barrier-derived biomarkers to also assess and monitor the clinical course of traumatic brain injuries in humans. Specifically, we here addressed the diffuse secondary disease progression of acceleration/deceleration injuries that is particularly difficult to capture [58]. Currently, blood–based biomarkers reflecting neuronal (i.e., ubiquitin carboxy-terminal hydrolase L1, UCH-L1) or astrocyte (i.e., glial fibrillary acidic protein, GFAP) have emerged, mostly differentiating more severe forms of TBI, but not (mild) concussion [59]. To this end, further analyses of the detailed microscopic and molecular damage of the neurovascular interface in the presented model might help derive biomarkers specific to concussion/CBI.

Notably, we only examined male mice. Several studies have described a sex-dependent difference in the severity and incidence of concussion in athletes [60]. Likewise, behavioral impairment after repetitive CBI, i.e., cued fear memory, was different in female compared with male mice [61]. The differential effect of sex and the potential influence of hormonal fluctuations need to be addressed in further studies.

Overall, we here introduce a preclinical model of CBI that constitutes a promising tool for translational investigation of concussion and its secondary complications, i.e., prolonged post-concussion syndrome and chronic traumatic encephalopathy. Different from other preclinical studies claiming to induce “mild TBI”, our model explicitly evokes a diffuse and multifocal BBB impairment corresponding to a characteristic neuro-psychiatric phenotype but without any evidence of focal cortical damage. Furthermore, our study comprises evidence that neuroimaging, as exemplified here by TSPO-PET, can detect early and disease-specific features that might be a valuable tool in longitudinal studies addressing individual vulnerability to secondary CBI-induced neurodegeneration.

## 4. Materials and Methods

### 4.1. Experimental Procedures

All animal procedures followed the German Laws for Animal Protection and were approved by the animal care committee and governmental authorities (Landesamt für Natur, Umwelt und Verbraucherschutz North Rhine-Westphalia, LANUV; AZ 81-02.04.2020.A058). Experiments were performed and reported in accordance with the ARRIVE guidelines. Animals were socially housed under a fixed 12 h light/dark cycle with ad libitum access to food and water. A total of 52 male C56BL/6J mice (either 7 or 11 weeks old, 21–26 g), were used for all experiments. All experimental procedures were performed either under anesthesia with isoflurane (4% for induction, 1.5–2% for maintenance in a 65%/35% nitrous oxide/oxygen atmosphere) supplied via a face mask, or in conscious sedation with medetomidine (0.1 mg/kg), as stated below. Mice received analgesia by tramadol (100 mg/mL) via drinking water, starting two days before and continuing for three days after CBI. Body temperature was measured via a fiber optic rectal probe (SA Instruments) and kept constant at 37 ± 1.0 °C by a water-circulating system (MedRes, Cologne, Germany).

### 4.2. Concussive Brain Injury (CBI)

For the establishment of the concussion model, a group of 16 mice received either a single concussion or sham surgery. CBI was induced by an adaptation of the controlled cortical injury model [11], but omitting prior opening of the skull. Instead, the closed skull was hit using a 5 mm impactor tip, softened by a custom-made silpuran cover (Figure 1A; ratio 1:1; stiffness 830 kPa). In detail, the anesthetized mice were positioned prone in a custom-made head holder made of polyethylene, to allow free rotational movement but at the same time limiting linear movements of the head (Figure 1B). Using a stereotactic frame, the impactor tip was lowered onto the exposed skull over the right primary motor cortex (AP +0.5 mm, ML 1 mm) in the protracted position, lightly touching the skull. After retraction, the tip was lowered by 3 mm (=penetration depth, dwell time = 0.1 s).

To minimize the impact of anesthesia on cognition, anesthesia was next switched to conscious sedation with the alpha agonist medetomidine at 0.1 mg/kg (Domitor®, Elanco, Greenfield, Indiana, USA, suspended in 250 µL of NaCl), and Isofluran was reduced to 0.5%. After 10 min, concussion was induced (impact velocity 5 mm/s, dwell time 0.1 s), and anesthesia immediately antagonized (Antisedan^®^, 0.5 mg/kg, Elanco, Greenfield, Indiana, USA, suspended in 250 µL of NaCl). The animals were returned to their home cages in a supine position, and the time until the first effort to rise from this position was measured as a surrogate marker to quantify loss of consciousness (LoC). Sham animals received the same treatment but instead of lowering, the impactor was raised by 3 mm, thus missing the animal on striking. Two animals died during surgery (one sham, one CBI) because of anesthesia-related complications.

To approximate the CBI-induced head rotation, experiments were filmed (GoProHero5, GoPro, San Mateo, CA, USA) at 240 frames per second. Head rotation was quantified by tracking of three well-visible fixpoints (eyes and nose) and calculated using Kinovea software (https://www.kinovea.org (accessed on 1 November 2023); vers. 0.9.4).

### 4.3. Behavioral Test

As a measure of the overall activity rate, nest-building activity was assessed. For this, animals received nestlet material of pre-defined weight, made of compacted paper (compact crinklets natural, SAFE^®^ Enrichment, Rosenberg, Germany). The unused (still compacted) material was weighed daily, and its reduction (i.e., the material used for nest-building activities), was normalized (%) to the original pellet.

Spontaneous locomotor activity and anxiety behavior were assessed five days after trauma using an open field cage (40 cm × 40 cm × 30 cm) equally lit from above with dimmed light (70–80 lux). Mice were allowed to freely explore the arena for ten minutes. Animal movement was automatically tracked (EthoVisionXt, Noldus, Weganingen, The Netherlands). For analyses, the arena was segmented into an outer “border” zone (Figure 2C, blue) and an inner “center” zone (Figure 2C, green). Analyses included the time spent in the border—resp. the center zone, as well as the overall distance covered. Additionally, we calculated an exploration ratio as time spent in the center zone relative to the border zone, using the formula (timecenter − timeborder)/(timecenter + timeborder).

To evaluate cognitive deficits, spontaneous alternation was measured using a Y-maze five days after CBI. Animals entered the Y-maze in its center, and their free exploration of the three arms was monitored for five minutes. The ratio of correct alternations, consisting of a consecutive visit to all three arms, was computed as a measure of cognition. Moreover, as an analysis of sensitivity, the novel object recognition test was additionally performed in a subgroup of mice. Four days after CBI, animals were familiarized with a first object (plastic bottle filled with blue liquid, Figure S1A right) for ten minutes within an open field arena described above. On the next day, animals were again placed in the arena equipped with the known object as well as a novel object (magic cube, Figure S1A left). The time spent with the novel relative to the known object (recognition ratio) was computed as (timenovel − timeknown)/(timenovel + timeknown).

In order to assess motor control, the rotating beam test was performed 1, 3, and 7 days after CBI. Mice were encouraged to run over a fiberglass beam of 120 cm in length and 13 mm in diameter, located 60 cm above a table covered with bubble cushions and rotating at 6 rpm. Mice were pre-trained three times before CBI, and the last trial was used as the baseline. The total time of traversal and the amount of foot slips were measured.

### 4.4. Repetitive CBI

To assess the impact of repetitive CBI, two different groups receiving five consecutive concussions were compared (Figure 3A). One group (blue arrows in Figure 3A, “repCBI-lf”) received one concussion every week, starting from the age of 7 weeks; the other group (orange arrows in Figure 3A, “repCBI-hf”) received one concussion every 48 h, starting at the age of 11 weeks. Both concussion groups were compared to animals receiving four sham surgeries and finally a single concussion (light grey arrow in Figure 3A, “CBI”), as well as to a sham group receiving 5 sham surgeries without any concussion (dark grey arrow in Figure 3A, “sham”).

Sample sizes were calculated a priori, based on the results of the prior exploratory trials, to yield 80% power to detect a significant cognitive impairment (Kruskal–Wallis Test) with a *p*-value < 0.05.

### 4.5. Structural Magnetic Resonance Imaging

To exclude gross structural damage, structural magnetic resonance imaging (MRI) was performed one day after the last concussion (black arrow in Figure 3A), using a small-animal 9.4 T horizontal MRI system (BioSpec; Bruker BioSpin, Ettlingen, Germany) with a 20 cm bore diameter and actively shielded gradient coils (BGA12S2, 600 mT/m; Bruker BioSpin). Radio frequency (RF) excitation and signal reception were performed with a 1H quadrature cryo-genic surface coil (CryoProbe, Bruker BioSpin). Image acquisition was accomplished using ParaVision 6.0.1 software (Bruker BioSpin). A T2-weighted spin echo (turbo-RARE, 24 slices, field of view (FOV) of 17.5 × 17.5 mm^2^, RARE factor = 8, repetition time (TR) = 5500 ms, echo time (TE) = 32.5 ms) sequence was performed and visually inspected.

### 4.6. PET Acquisition and Image Analysis

Positron emission tomography (PET) using [^18^F]DPA-714, a second-generation TSPO ligand, was conducted in six animals per group, six days after the last concussion (red arrow in Figure 3A). The tracer was synthesized as described previously [62]. Prior to tracer application, animals were anesthetized with isoflurane, and a catheter for tracer injection was inserted into the lateral tail vein. Mice were placed in a custom-made animal holder (medres^®^ GmbH, Cologne, Germany) with the head fixed in a tooth bar. Scans were conducted in list mode using a Focus 220 micro-PET scanner (CTI-Siemens, Erlangen, Germany; resolution at the center of field of view: 1.4 mm). Data acquisition over 30 min started immediately after intravenous tracer injection (activity: 8–12 MBq in 125 µL). A 10-min transmission scan using a 57Co point source was conducted for attenuation correction. After imaging, the catheters were removed, and the mice were returned to their home cages.

After full 3D rebinning, summed images were reconstructed (voxel size: 0.47 ×  0.47 × 0.80 mm). VINCI 4.72 for Windows (Max Planck Institute for Metabolism Research, Cologne, Germany) was used for further analyses. After co-registration, images were intensity-normalized to a background signal. To this end, an elliptical volume of interest (VOI) of 4 mm^3^ was placed inside a background region within the midbrain of each individual image. Each image was divided by the mean value of the background VOI, resulting in the “background-standardized uptake value ratio” (SUVbg). Mean intensity values within a hippocampus mask, separated for each hemisphere, were used for further analyses. Additionally, time–activity curves were compared between groups (Figure S1D).

### 4.7. Histology and Immunofluorescence

Seven days after the last concussion, animals were deeply anesthetized and systemically perfused with 20 mL ice-cold phosphate-buffered saline (PBS, Gibco™ PBS, Thermo Fisher Scientific, Darmstadt, Germany) and subsequent endovascular fixation with 20 mL paraformaldehyde (PFA, ROTI^®^Histofix 4%, Carl Roth GmbH + Co. KG, Karlsruhe, Germany) infused constantly at 9 mL/min. Brains were immediately shock-frozen in methylbutane at −60 °C. All staining techniques were performed on coronal brain slices of 10 µm thickness.

For assessment of structural damage, hematoxylin and eosin (H&E) staining was performed. Slides were stained for 20 min with Mayer’s hemalaum solution (Mayers Hämalaunlösung 109249, Merck^®^, Rahway, NJ, USA), differentiated in ethanol (70%) before staining with eosin (Eosin G-Lösung 0.5%, Carl Roth^®^, Karlsruhe, Germany) for 3 min, and subsequently washed with distilled water. Afterward, the slices were dipped for 2 min each in an ascending alcohol series and coverslipped with Entellan.

Immunoglobulin G (IgG) extravasation was examined by staining for mouse IgG. After antigen retrieval using 0.3% H_2_O_2_, slices were incubated with 1% normal horse serum (NHS) for one hour to prevent unspecific antibody binding. Biotinylated anti-mouse IgG (BA-2000, Vector Laboratories, Newark, NJ, USA) antibody was applied (1:500 in PBS) for 1.5 h. Antibody binding was visualized by an avidin/biotin-based peroxidase system (Vectastain ABC kit, Vector Laboratories) and the chromogen diaminobenzidine (DAB). One experimental group of eight mice was stained at the same time by an investigator blinded to group allocation. From the region of interest, mean color intensities were assessed using MATLAB (2020b, MathWorks, Natick, MA, USA). Because of the different coloration across trials, Z-transformation was performed.

To visualize microglia and astrocytes, staining for Iba-1 and GFAP were performed, respectively. Additional post-fixation in PFA for 30 min was performed before antigen retrieval by incubation in citrate buffer (pH = 6) at 80 °C for 20 min. To prevent unspecific antibody binding, slices were incubated with 5% donkey serum and 0.25% Triton X (Tx) in PBS for one hour. Primary antibodies (Iba1, rabbit monoclonal antibody, 1:200, ab178847, Abcam, Cambridge, U.K.; GFAP, mouse monoclonal antibody, 1:200, MAB360, Sigma) were incubated in 1% donkey serum, 0.25% Tx, and PBS overnight before incubation with a secondary antibody (Alexa Fluor donkey anti-rabbit IgG or Alexa Fluor donkey anti-mouse, 1:500, Invitrogen, Karlsruhe, Germany) diluted in 1% NDS and 0.25% Tx in PBS. Slices were counterstained with Hoechst (1:500) for 10 min. Images of the hippocampus were acquired using an inverted fluorescence microscope (BZ 9000, Keyence, Osaka, Japan), with a 40× magnification, and Iba-1 and GFAP-positive microglia were counted manually by a blinded investigator.

### 4.8. Dextran Extravasation

To detect blood–brain barrier disruption at various timepoints, mice receiving five repetitive CBIs (“repCBI-hf”) were sacrificed either one, three, or seven days after the last trauma compared to a group of mice receiving sham surgeries. Under low-light conditions, mice were perfused with Texas-red labeled dextran (40 kDa, D1829, Invitrogen, Waltham, MA, USA) solved in saline containing heparin [63]. Brains were extracted and shock-frozen in methylbuthane. Using a cryostat, 30 µm thick coronal slices across the hippocampus were cut. In addition to counterstaining with Hoechst as described above, capillaries were stained using Lectin (Lycopersicon Esculentum (Tomato) Lectin, DyLight 488, 1:400, Invitrogen L32470). Sections were imaged using a confocal microscope (TCS SP8, Leica, Wetzlar, Germany).

A region of interest (ROI) outlining the hippocampus was manually drawn. Using a custom script written using MATLAB (2020b, MathWorks, Massachusetts), a mask was generated based on the Lectin-positive capillaries (Figure S1G) to mask out intravascular dextran signal (Figure S1H). The remaining images were used to compute histograms of intensity values across the red color channel (RGB brightness intensities between 0 and 255) and its mean intensity values were compared across groups.

### 4.9. Statistical Analyses

Data normality was assessed by visualization of histograms and analysis of the Kolmogorov–Smirnov test. For analyses of differences across groups, a One-way ANOVA or Kruskal–Wallis test was used in the case of normally or not normally distributed data, respectively. For analysis of repetitively measured behavioral data, a mixed ANOVA with the kind of trauma applied as a between-group factor and time as a within-group factor was modeled. Spearman correlation was used to analyze associations between features. A *p*-value of less than 0.05 was considered significant.

## Data Availability

As part of an ongoing investigation, all data that support the findings of this study and all custom-written MATLAB codes are available from the corresponding author upon reasonable request.

## Supplementary Materials

The supporting information can be downloaded at: https://www.mdpi.com/article/10.3390/ijms25094837/s1.

## Abbreviations

BBB	blood-brain barrier
CBI	concussive brain injury
GFAP	glial fibrillary acidic protein
IgG	immunoglobulin G
LoC	loss of consciousness
MRI	magnetic resonance imaging
PET	positron emission tomography
repCBI-hf	repetitive CBI—high frequency
repCBI-lf	repetitive CBI—low frequency
SUV	standard uptake values
TBI	traumatic brain injury
TSPO	translocator protein
VOI	volume of interest
