# Supplementary material for: Early Blood–Brain Barrier Impairment as a Pathological Hallmark in a Novel Model of Closed-Head Concussive Brain Injury (CBI) in Mice
Source: Int J Mol Sci. 2024 Apr 29;25(9):4837. doi: 10.3390/ijms25094837 (PMC11084321; doi:10.3390/ijms25094837)
Supplement: Supplementary file 1 [file ijms-25-04837-s001.zip › ijms-2957124-supplementary.pdf]

Supplementary Figures

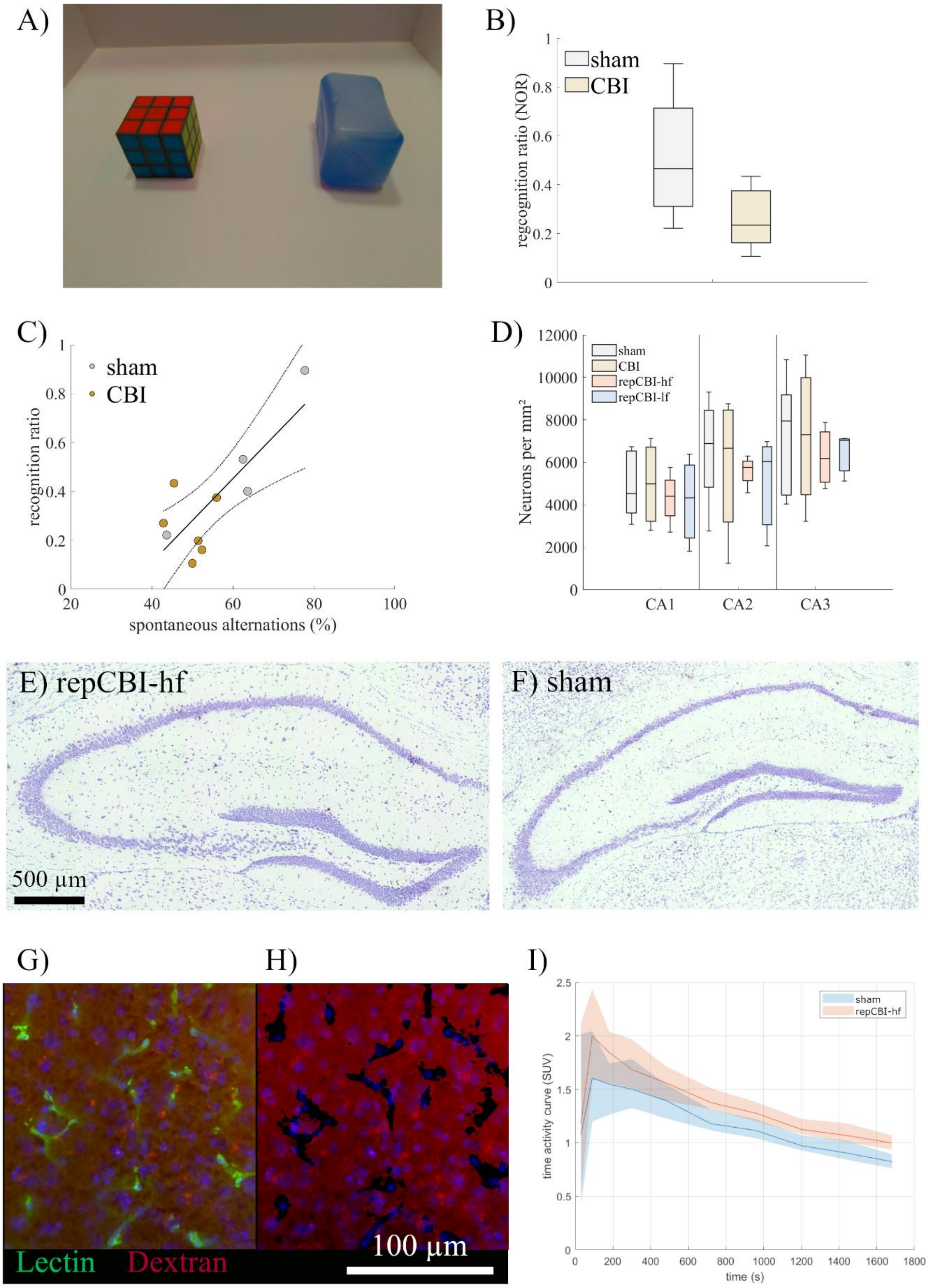

Suppl. Figure S1:

To corroborate our findings of spontaneous alternations using the Y-maze test, we additionally investigated novel object recognition (NOR; **A**) using a familiar object (blue cube, right) and a new object (magic cube, left). Here, mice after CBI showed a similar decrease in recognition memory (**B**), showing a good correlation with impairments in spatial cognition (**C**).

To assess neuronal impairment, hippocampal slides were stained using cresylviolett. Neither group of repetitive (**E**) or single CBI demonstrated signs of neuronal impairment compared to the sham group (**F**). Additionally, density of neurons (per mm^2) were counted in different regions of the cornu ammonis (CA 1-3, **D**).

In order to measure immunoglobuline extravasation, slices after dextran perfusion were counterstained by Lectin (**G**, green). Using these images, a mask of Lectin-labeled capillaries was thresholded und masked out from the dextran image (**H**). The residual image was used for calculation of dextran intensity.

To further explore the found increase in [18F]DPA-714 after CBI, time activity curves (TAC) of tracer accumulation within the hippocampus were explored (**I**), revealing no significant differences in the temporal tracer dynamics.
